# Investigation of Polyaniline and a Functionalised Derivative as Antimicrobial Additives to Create Contamination Resistant Surfaces

**DOI:** 10.3390/ma11030436

**Published:** 2018-03-16

**Authors:** Julia Robertson, Marija Gizdavic-Nikolaidis, Simon Swift

**Affiliations:** 1 Department of Molecular Medicine and Pathology, The University of Auckland, Auckland 1023, New Zealand; julia.robertson@auckland.ac.nz; 2 School of Chemical Sciences, The University of Auckland, Auckland 1142, New Zealand; m.gizdavic@auckland.ac.nz

**Keywords:** antimicrobial, surfaces, infection control, polyaniline, *Escherichia coli*, *Staphylococcus aureus*

## Abstract

Antimicrobial surfaces can be applied to break transmission pathways in hospitals. Polyaniline (PANI) and poly(3-aminobenzoic acid) (P3ABA) are novel antimicrobial agents with potential as non-leaching additives to provide contamination resistant surfaces. The activity of PANI and P3ABA were investigated in suspension and as part of absorbent and non-absorbent surfaces. The effect of inoculum size and the presence of organic matter on surface activity was determined. PANI and P3ABA both demonstrated bactericidal activity against *Escherichia coli* and *Staphylococcus aureus* in suspension and as part of an absorbent surface. Only P3ABA showed antimicrobial activity in non-absorbent films. The results that are presented in this work support the use of P3ABA to create contamination resistant surfaces.

## 1. Introduction

Microbial resistance to antimicrobial agents is increasing worldwide and it represents a major threat to the successful treatment of infectious diseases [1]. Development of antimicrobial resistance is an inescapable consequence of natural selection and is associated with exposure to antimicrobial agents [1]. Efforts need to be made to decrease the unnecessary exposure of bacteria to antibiotics to reduce the selective pressure driving the development of resistance so that existing antibiotics retain their efficacy for as long as possible [1]. In part, this can be achieved by controlling the spread of pathogenic bacteria and therefore reducing the number of infections that require antibiotic treatment [2].

Healthcare-associated infections are a major contributor to patient morbidity and mortality, and occur in part due to bacterial contamination of hospital surfaces [3]. Surfaces in hospitals that come into contact with hands are regularly contaminated with nosocomial pathogens [4,5]. Infected patients shed pathogenic bacteria, including methicillin-resistant *Staphylococcus aureus* and vancomycin-resistant *Enterococcus* spp., into their immediate environment [5,6,7,8,9,10]. Surfaces near shedding patients, such as walls, door handles, bed frames, and light switches, tend to be touched frequently and therefore are more likely to be contaminated [3,10,11]. Once a surface is contaminated, a single hand contact event is sufficient to transmit bacteria from the surface to a person [7,8,12].

Bacteria that have been transferred to a surface can persist for a period of time or actively colonise to form a biofilm. Bacterial persistence on a surface is influenced by dynamic environmental conditions, including organic soiling, humidity, and temperature [4,13,14]. Biofilms are highly recalcitrant to antimicrobial treatments and facilitate the persistence of bacteria on surfaces resulting in surface associated pathogen reservoirs, which increase the risk of transmission [15,16,17]. The level of bacterial transfer that occurs between a contaminated surface and a hand following contact has been demonstrated to occur at a comparable level to direct contact with an infectious patient, which is a well-established transmission route [4,18,19]. Hand washing can help to control the spread of infection in hospitals; however, without the decontamination of surfaces, the reservoirs of pathogens will seed further spread. Nosocomial pathogens isolated from hospital surfaces are typically in the range of 100–10,000 colony forming units (CFU)/cm^2^ [5,10]. For a microbial burden exceeding 250 CFU/100 cm^2^, transmission from the surfaces to health care workers and/or patients increases [10,20,21]. Therefore, despite the relatively low inocula present, any contamination of a hospital surface by a pathogen should be considered to be a transmission risk [10,20,21].

The involvement of contaminated surfaces in pathogen transmission pathways in hospitals necessitates the improved control of surface microbiology. Reduction of microbial contamination on hospital surfaces could disrupt transmission pathways and potentially reduce infectious disease incidence rates and the associated antibiotic usage [22]. Utilisation of antibacterial surfaces is a promising means of reducing microbial surface load as well as preventing formation of biofilms and surface associated pathogen reservoirs [2]. An ideal antimicrobial surface would be active against relevant bacteria at appropriate bacterial loads and active in environmental conditions relating to potential applications in terms of temperature, relative humidity, pH, exposure to cleaning products, and contaminating organic matter [23,24,25]. The time that is required for decontamination would need to be sufficiently short to be effective in breaking transmission pathways. Activity needs to be retained for sufficiently long periods of time, and after repeated bacterial challenges, to be cost effective [26,27]. Activity overtime is informed by whether the antimicrobial agent is immobilised on the surface or if it has to be released to elicit an effect [28,29]. The release of an antimicrobial agent over time means that the surface concentration of the agent will fall below the threshold needed to exert antimicrobial activity [27]. An ideal antimicrobial surface would also need to be cheap and easy to make, suitable for large-scale production, and have regulatory approval for the intended use [26,30].

To create an antimicrobial surface, we can take one of two basic approaches. First, a coating may be applied to a material or a modification of the surface chemistry of the material made to provide an antimicrobial surface [31]. Alternatively, the material may be fabricated by incorporating an antimicrobial into the material, which can be challenging as manufacturing procedures can involve extreme environmental conditions, including high temperatures and shear forces, which can negatively impact on bactericidal activity [26,28,32]. Covalent attachment of an antimicrobial agent to a surface may cause side reactions that result in conformational changes in the agent, ultimately causing a loss of activity [33]. Therefore, the method of antimicrobial surface production may affect the resulting surface activity.

The activity of an antimicrobial surface is also influenced by the nature of the surface. Surfaces can be absorbent allowing water droplets to move into the surface or they can be non-absorbent, in which water droplets sit on top of the surface [34,35]. These surface properties may affect the antimicrobial activity as a bacterium in a water droplet would have more contact with the antimicrobial agent if it has absorbed into the surface. Non-absorbent surfaces in hospitals are frequently contaminated with pathogens, and include walls, door handles, and bed frames. Much of the focus of development of antimicrobial surfaces in the published literature is on model non-absorbent surfaces, such as metal coupons and plastic films [36,37,38]. Many absorbent surfaces in hospitals are fabric-based, such as apparel worn by healthcare workers and patient privacy curtains [36,39,40]. Privacy curtains are high-touch areas that are contacted by the hands of the healthcare worker before, during, and after patient care, and are infrequently changed [40,41]. It has been demonstrated that more than 90% of privacy curtains can become contaminated within a week of use [41]. Contaminated absorbent surfaces in hospitals may be involved in pathogen transmission [36,39]. Absorbent surfaces are harder to clean or disinfect than non-absorbent surfaces, while the latter facilitates a greater transfer of bacteria [8,41,42]. Therefore, the development of both absorbent and non-absorbent antimicrobial surfaces would help to curtail the spread of infection in hospitals [1].

Antimicrobial polymers are good candidates for immobilised biocides. These polymers can be either polymeric biocides (the repeating unit is a biocide) or biocidal polymers (the active principle is embodied by the whole macromolecule) [28,29]. In this article, we investigate the antimicrobial activity of polyaniline (PANI) and a functionalised derivative (fPANI), homopolymer poly(3-aminobenzoic acid) (P3ABA), as surface-immobilised biocidal polymers. Utilisation of PANI for potential applications is restricted because of its insolubility in common solvents, which renders it difficult to process [43,44]. fPANIs are easily and inexpensively synthesised using substituted aniline monomers, which improves the solubility, and thus the processability, of the resulting polymer [43,44]. PANI and P3ABA are good candidates for incorporation into surfaces because they have thermal stability up to 300 °C, environmental stability in the conducting form, simple and inexpensive synthetic procedures [45,46,47,48], and have been demonstrated to be biocompatible with mammalian cells [49,50,51,52], all of which increases their commercial viability. Surfaces containing PANI and P3ABA are non-leaching [45,53,54], which promotes activity over a longer period of time and reduces both personal and environmental safety concerns [27].

In this study, we investigated the potential of PANI and P3ABA as surface antimicrobial agents. Initial testing involved the challenge of target organisms in suspension, mirroring standard antimicrobial susceptibility testing methods [55,56]. The target organisms selected were the antimicrobial susceptibility testing strains, *Escherichia coli* 25922 and *S. aureus* 6538, which reflect bacteria that are commonly isolated from surfaces in hospitals [10,57]. Susceptibility to antimicrobial activity can be influenced by media composition through its effects on bacterial cell physiology [58]. Therefore, *E. coli* was challenged in Lennox Broth (LB)—a rich media on which cells grow at high rates—and in minimal A salts with 0.4% succinate as the carbon source, which only contains nutrients that are essential for growth [59,60,61]. The slow growth of bacteria in a minimal media environment is similar to what may occur on surfaces in nature [62].

**Figure 1:**
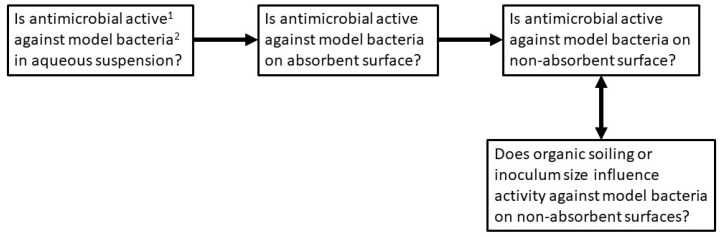
Experimental strategy. Polyaniline (PANI) and functionalised derivative (fPANI) are tested according to the scheme presented. ^1^ Activity is measured as reduction in the number of viable cells recovered from surfaces. ^2^ The Gram positive bacterium *S. aureus*, and the Gram-negative bacterium *E. coli* were tested as model species.

Following confirmation of activity in suspension, PANI and P3ABA were incorporated into absorbent and non-absorbent surfaces. The effect of incorporation on antimicrobial activity was determined in 96 well plate based assays, which allowed for the the testing of many concentrations and treatment times against one inoculum [27]. Absorbent surfaces were modelled using agar mixed with varying amounts of PANI or P3ABA [63]. Drops of liquid containing bacteria absorbed into the solidified agar test surfaces [64]. Non-absorbent surfaces were established in the form of compression moulded Styrene Ethylene Butylene Styrene (SEBS) films [63]. The activity of non-absorbent surfaces containing PANI and P3ABA were then characterised in relevant environmental conditions, including challenging with a range of inocula and in the presence of organic matter [62]. The experimental strategy is summarised in Figure 1, and, taken together, the results presented demonstrate the activity of PANI and P3ABA in suspension and in surfaces, in application relevant settings. The efficacious activity of P3ABA supports the utilisation of this polymer to create contamination resistant surfaces.

## 2. Results

### 2.1. Activity of PANI and P3ABA Suspensions against E. coli and S. aureus

To examine the activity of PANI and P3ABA, cell viability assays were performed on *E. coli* 25922 *lux* and *S. aureus* 6538 challenged with 0.5% (*w*/*v*) suspensions. Activity was determined in rich media, LB broth, for *E. coli* and *S. aureus,* as well as a minimal media, minimal A salts with succinate as the carbon source, for *E. coli*. At 0.5 h, 1 h, 2 h, and 4 h time points the treated cells were enumerated using the drop count method [64].

**Figure 2:**
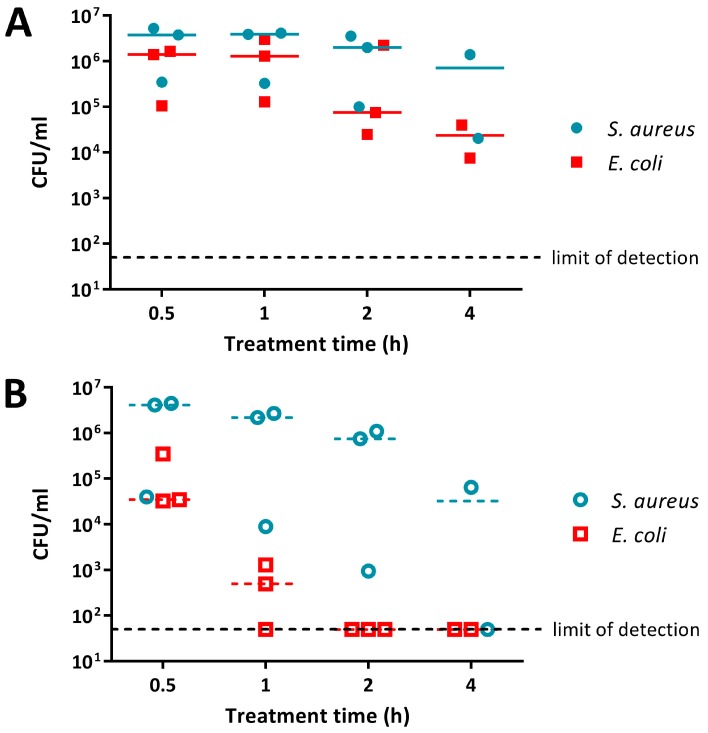

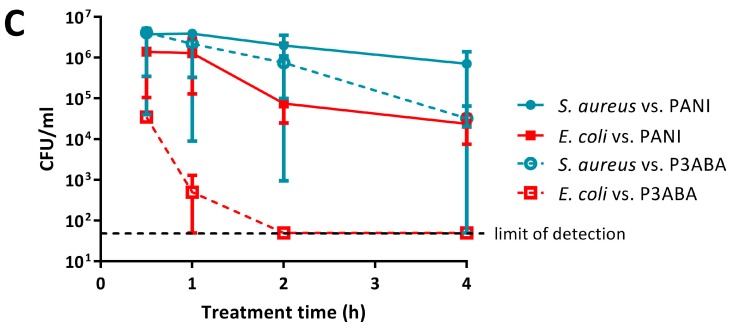
Sensitivity of *E. coli* 25922 *lux* and *S. aureus* 6538 to PANI and poly(3-aminobenzoic acid) (P3ABA) suspensions. Cell viability assays of ~10^6^ CFU/mL *E. coli* and *S. aureus* treated with 0.5% PANI suspension (**A**) and 0.5% P3ABA suspension (**B**) in Lennox broth (LB), with each symbol representing the median of three technical replicates and the bar representing the median of each biological replicate. The data in A and B is replotted together in (**C**) where each point represents the median of biological replicates and the error bars are the range Viable cell counts (colony forming units (CFU)/mL) were obtained for each strain at 0.5 h, 1 h, 2 h, and 4 h time points. The limit of detection is 50 CFU/mL.

*E. coli* and *S. aureus* treated with PANI suspension were present at similar cell numbers at the earlier, 0.5 h and 1 h, time points (Figure 2A). For the later, 2 h and 4 h, time points *E. coli* was knocked down by 1 to 2 logs (measured as the difference between inoculum and the median number of viable cells remaining), while *S. aureus* cell numbers decreased by only ~0.5 log (Figure 2A). The overall difference in sensitivity between *E. coli* and *S. aureus* to 0.5% PANI suspension was statistically significant (linear regression analysis, intercepts are different, *p* value: less than 0.05).

Both *E. coli* and *S. aureus* were more susceptible to P3ABA suspension when compared to PANI suspension (Figure 2). P3ABA suspension reduced *E. coli* viable cell numbers to below the limit of detection within 2 h, while *S. aureus* was knocked down by ~2 log following a 4 h exposure (Figure 2B; i.e., the difference between inoculum and the median number of viable cells remaining was about 2 logs, or 100-fold). As observed for PANI suspension, P3ABA suspension was more active against *E. coli* as compared to *S. aureus* (Figure 2B,C). The difference in susceptibility between *E. coli* and *S. aureus* to 0.5% P3ABA suspension was statistically significant (linear regression analysis, intercepts are different, *p* value: less than 0.05). These results confirm that PANI and P3ABA in suspension are active against the model Gram-negative and Gram-positive organisms tested and support investigation of their surface activity.

**Figure 3:**
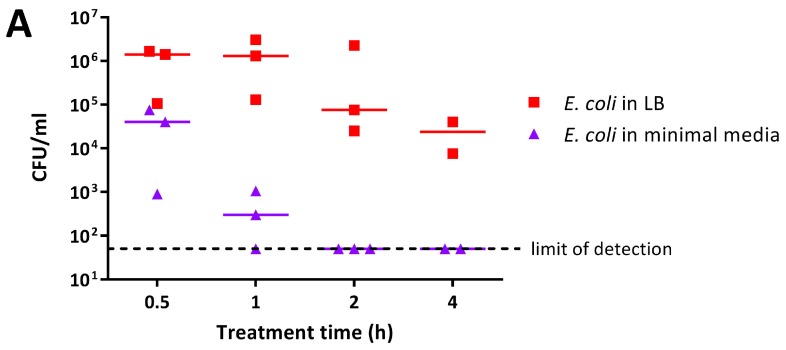

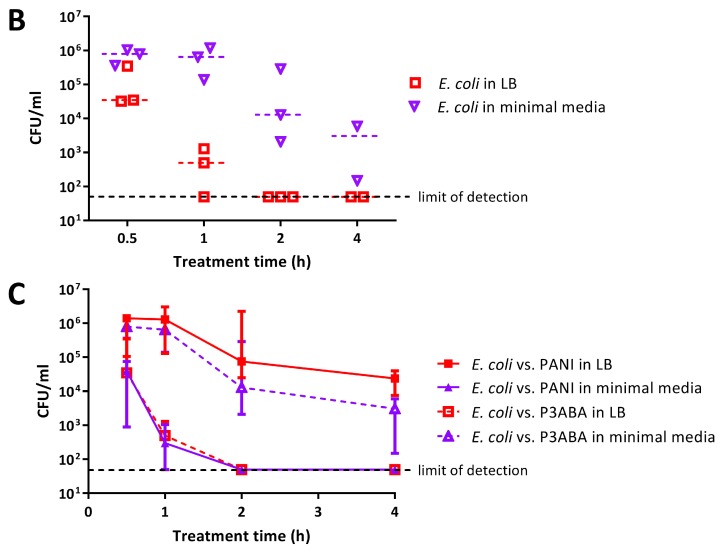
Sensitivity of *E. coli* 25922 *lux* to PANI and P3ABA suspensions in rich and minimal media. Cell viability assays of ~10^6^ CFU/mL *E. coli* treated with 0.5% PANI suspension (**A**) and 0.5% P3ABA suspension (**B**) in Lennox broth (LB) and minimal A salts, with each symbol representing the median of three technical replicates and the bar representing the median of each biological replicate. The data in A and B is replotted together in (**C**) where each point represents the median of biological replicates and the error bars are the range. Viable cell counts (CFU/mL) were obtained for experimental sample at 0.5 h, 1 h, 2 h, and 4 h time points. The limit of detection is 50 CFU/mL.

The antimicrobial activity of 0.5% PANI and P3ABA suspensions in rich and minimal media was determined against *E. coli* 25922 *lux*. PANI suspension mediated a greater reduction in the viable cell count in minimal media when compared to rich media (Figure 3A). *E. coli* in minimal media reached the limit of detection (~4 log reduction) after a 2 h challenge, while a decrease of ~1–2 log was observed in rich media after 4 h (Figure 3A). The greater activity of PANI suspensions against *E. coli* in minimal media when compared to LB broth was statistically significant (linear regression analysis; intercepts are different, *p* value: less than 0.05).

P3ABA suspension was more active against *E. coli* in rich media as compared to minimal media (Figure 3B). *E. coli* in LB broth was knocked down ~3 log after 1 h and reached the limit of detection by 2 h (Figure 3B). In comparison, the levels of viable *E. coli* in minimal media were stable at the 0.5 h and 1 h time points (Figure 3B). After 4 h of treatment, P3ABA knocked down *E. coli* in minimal media by ~2 log (Figure 3B). The difference in activity of P3ABA against *E. coli* in LB broth and *E. coli* in minimal media was statistically significant (linear regression analysis, slopes are different, *p* value: less than 0.05). These results confirm that PANI and P3ABA in suspension are active against *E. coli* 25922 in rich and minimal media, and support the investigation of their surface activity.

### 2.2. Activity of Absorbent Surfaces Containing PANI and P3ABA against E. coli and S. aureus

Following the demonstration of activity for PANI and P3ABA against *E. coli* and *S. aureus*, we investigated the antibacterial activity of absorbent surfaces containing PANI and P3ABA to simulate surfaces that absorb water droplets, such as fabrics [65]. LB agar as used as a model of an absorbent surface. Agar containing 1% and 2% concentrations of PANI and P3ABA in agar were tested along with a higher concentration of PANI (8% PANI), because PANI was less active than P3ABA in suspension (Figure 2).

**Figure A1:**
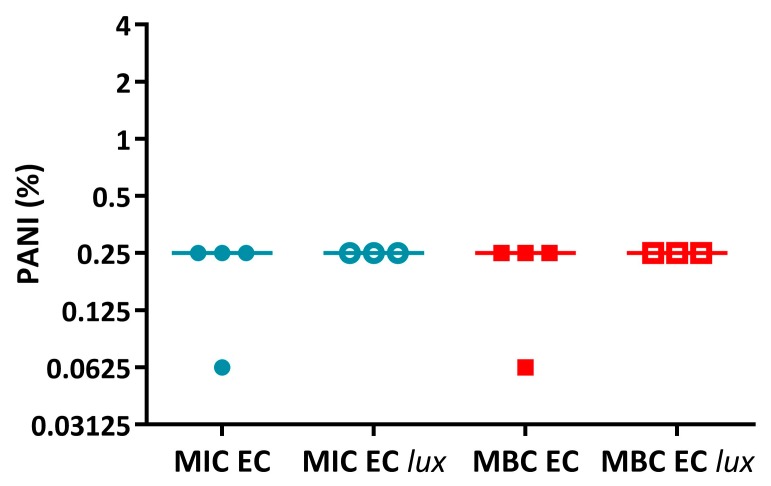
Activity of PANI against *E. coli* 25922 and *E. coli* 25922 *lux*. The MIC (circles) and MBC (squares) of PANI against *E. coli* 25922 (EC) and *E. coli* 25922 *lux* (EC *lux*) in LB broth. Data obtained from *E. coli* 25922 is represented by filled data points while data obtained from *E. coli* 25922 *lux* is represented by unfilled data points.

**Figure A2:**
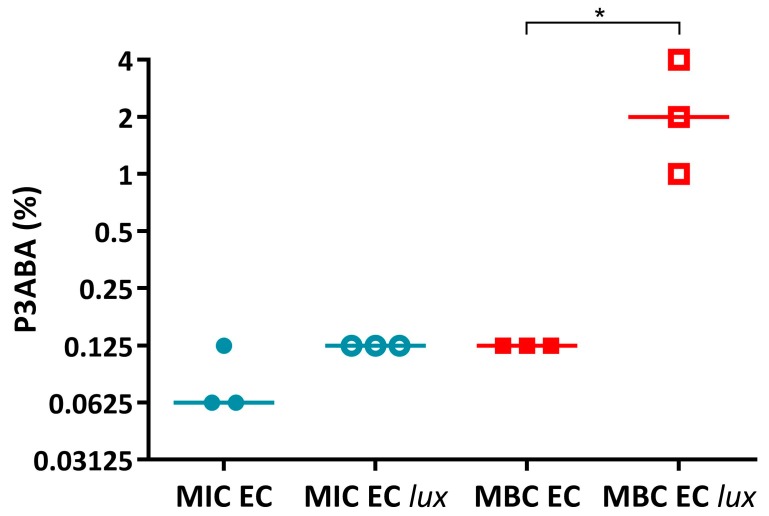
Activity of P3ABA against *E. coli* 25922 and *E. coli* 25922 *lux.* The MIC (circles) and MBC (squares) of PANI against *E. coli* 25922 (EC) and *E. coli* 25922 *lux* (EC *lux*) in LB broth. Data obtained from *E. coli* 25922 is represented by filled data points while data obtained from *E. coli* 25922 *lux* is represented by unfilled data points. Statistical significance is represented by * (Mann-Whitney test, *p* value: less than 0.05).

To investigate antimicrobial activity, *E. coli* 25922 *lux* or *S. aureus* 6538 were inoculated onto the experimental agar, with cells being rescued at various time points in fresh media. Survival of the PANI or P3ABA in agar challenge was based on growth of rescued cells. A *lux*-tagged version of *E. coli* 25922 was used in this work as bioluminescence is a practical alternative to enumeration by plate counts [66,67,68] (Appendix A, Figure A1 and Figure A2). Growth of *E. coli* 25922 *lux* was determined by measuring bioluminescence using the VICTOR X Multilabel Plate Reader. Growth of *S. aureus* 6538 was determined by examining optical density at 600 nm (OD_600_) using the µQuant™ Microplate Spectrophotometer as a *lux*-tagged *S. aureus* 6538 strain was not available for testing.

**Figure 4:**
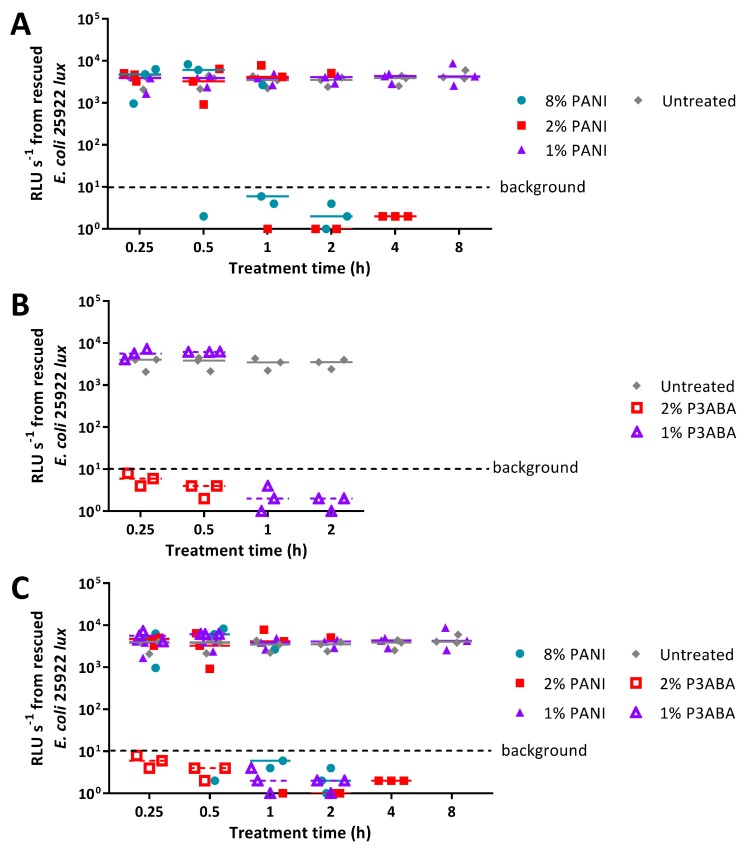
Sensitivity of *E. coli* 25922 *lux* to PANI and P3ABA in agar. (**A**) ~10^4^ CFU of *E. coli* was exposed to 8% PANI, 2% PANI, and 1% PANI incorporated into LB agar for 0.25 h, 0.5 h, 1 h, 2 h, 4 h, and 8 h. (**B**) ~10^4^ CFU of *E. coli* was exposed to 2% P3ABA and 1% P3ABA incorporated into LB agar for 0.25 h, 0.5 h, 1 h, and 2 h. Following treatment, the cells were rescued by washing the agar surface with LB broth and transferred to a 96 well plate. Each point represents the median of three technical replicates and each bar represents the median of each biological replicate. The data from A and B is combined for comparison in (**C**). The rescued cells were incubated at 37 °C for 16 h and light release was measured. The vertical axis shows the bioluminescence measurements (relative light units per second, RLU s^−1^) from the recovered cells with each data point representing an independent experiment and the line representing the median. Background luminescence readings are ~10 RLU s^−1^.

**Figure A3:**
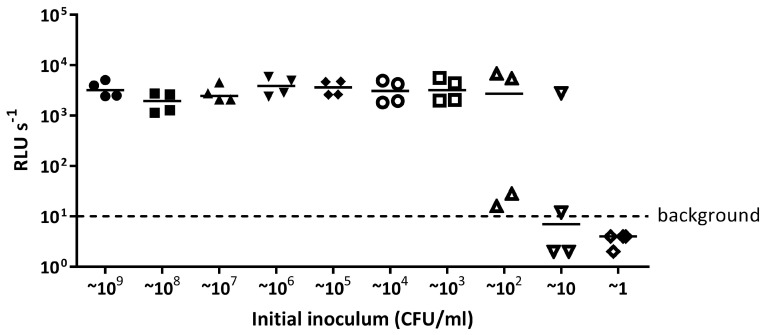
The limit of detection of *E. coli* 25922 *lux* when grown in a 96 well plate. *E. coli* 25922 *lux* was serially diluted in LB broth from ~10^9^ CFU/mL to ~1 CFU/mL in a 96 well plate and incubated at 37 °C for 16 h. Light release (RLU s^−1^) was measured from each dilution and detectable light levels above 10 RLU s^−1^ indicates growth. The data presented are from four independent experiments.

Agar containing 8% PANI mediated a decrease in bioluminescence levels of *E. coli* 25922 *lux* to background levels after 1 h of treatment, while 2% PANI agar required 2 h to reduce surface bacterial load (Figure 4A). The limit of detection for this assay is ~20 CFU (Figure A3). Agar containing 1% PANI did not exhibit antimicrobial activity against *E. coli* (Figure 4A). P3ABA agar had greater surface antimicrobial activity than PANI agar (Figure 4). Agar containing 1% P3ABA and 2% P3ABA reduced bioluminescence levels from *E. coli* 25922 *lux* to background levels within 1 h and 15 min, respectively (Figure 4B).

**Figure 5:**
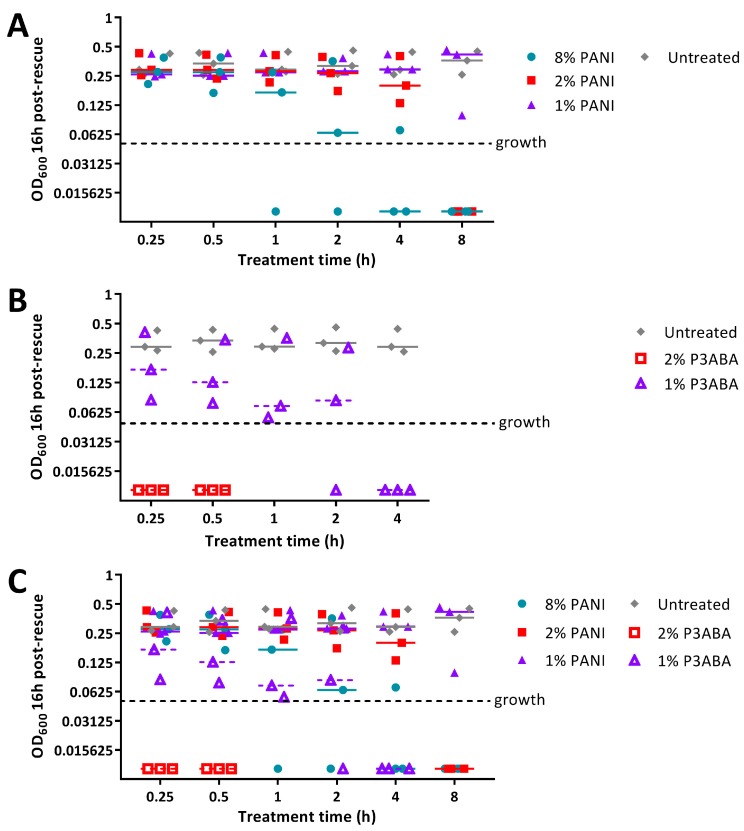
Sensitivity of *S. aureus* 6538 to PANI in agar. (**A**) ~10^4^ CFU of *S. aureus* was exposed to 8% PANI, 2% PANI, and 1% PANI incorporated into LB agar for 0.25 h, 0.5 h, 1 h, 2 h, 4 h, and 8 h. (**B**) ~10^4^ CFU of *S. aureus* was exposed to 2% P3ABA and 1% P3ABA incorporated into LB agar for 0.25 h, 0.5 h, 1 h, 2 h, and 4 h. Following treatment, the cells were rescued by washing the agar surface with LB broth and transferred to a 96 well plate. Each point represents the median of three technical replicates and each bar represents the median of each biological replicate. The data from A and B is combined for comparison in (**C**). The rescued cells were incubated at 37 °C for 16 h and optical density at 600 nm (OD_600_) was measured. The vertical axis shows the OD_600_ measurements from the recovered cells with each data point representing an independent experiment and the line representing the median. OD_600_ readings above 0.05 are considered as growth.

**Figure A4:**
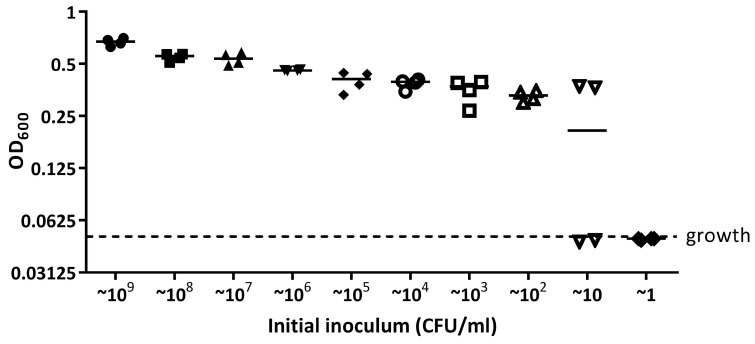
The limit of detection of *S. aureus* 6538 when grown in a 96 well plate. *S. aureus* 6538 was serially diluted in LB broth from ~10^9^ CFU/mL to ~1 CFU/mL in a 96 well plate and incubated at 37 °C for 16 h. OD_600_ was measured from each dilution and OD_600_ readings above 0.05 are considered as growth. The data presented are from four independent experiments.

PANI in agar was less active against *S. aureus* 6538 than *E. coli* 25922 *lux*, reflecting the trend observed with suspension testing (Figure 2A, Figure 4A, and Figure 5A). Agar containing 2% and 8% PANI reduced *S. aureus* surface load to background levels within 8 h and 4 h, respectively (Figure 5A). The limit of detection for this assay is ~20 CFU (Figure A4). As observed for *E. coli*, 1% PANI in agar was inactive against *S. aureus* within the time constraints (8 h) of the experiment (Figure 4A and Figure 5A). The viability of *S. aureus* cells following treatment with 8% PANI in agar for 2 h, 4 h, and 8 h, and following treatment with 2% PANI in agar for 8 h was significantly different from that of untreated cells (Friedman test, *p* value: less than 0.05, Dunn’s multiple comparison test).

Agar containing 2% P3ABA effectively decontaminated *S. aureus* on a surface following a 15 min treatment (Figure 5B), which was consistent with the activity against *E. coli* (Figure 5A). 1% P3ABA in agar was less active than the higher concentration, with the former requiring a 4 h exposure to reduce bacterial load (Figure 5B). The activity of 2% P3ABA in agar over 15 min and 1% P3ABA in agar over 4 h was statistically significant (Friedman test, *p* value: less than 0.05, Dunn’s multiple comparison test).

The activity of PANI and P3ABA presented in Figure 4 and Figure 5 demonstrates their efficacy against *E. coli* and *S. aureus* when incorporated into an absorbent surface.

### 2.3. Activity of Non-Absorbent Surfaces Containing PANI and P3ABA against E. coli and S. aureus

The activity of PANI and P3ABA as surface antimicrobials at non-absorbent surfaces to simulate surfaces that do not absorb water, such as walls and door handles, was investigated using SEBS films containing 5% PANI or 3% P3ABA [69]. The concentrations of the additive in these films are within the range typically used for incorporation into surfaces (0.1–5%) [69] and reflect the greater activity of P3ABA against *E. coli* and *S. aureus* compared to PANI as demonstrated in suspension (Figure 2) and in agar (Figure 4 and Figure 5). The activity of PANI and P3ABA films was examined in a ‘micro-surface testing assay’ (MSTA), in which 10 µL of inoculum in LB broth is sandwiched between two pieces of film and recovered at particular time points in fresh LB broth in a 96 well plate [28,70]. Cell viability was determined by measuring the bioluminescence for *E. coli* 25922 *lux* and OD_600_ for *S. aureus* 6538.

The MSTA for testing 5% PANI and 3% P3ABA films against bacteria was optimised using *E. coli* 25922 *lux*. Following either 2 h or 24 h challenges on the films, bacteria were rescued by washing with LB broth and incubated in a 96-well plate for 16 h, after which bioluminescence was measured. The bacteria present in the remaining recovery broth were enumerated using plate counts to verify the ability of bioluminescence levels to infer cell number.

**Figure 6:**
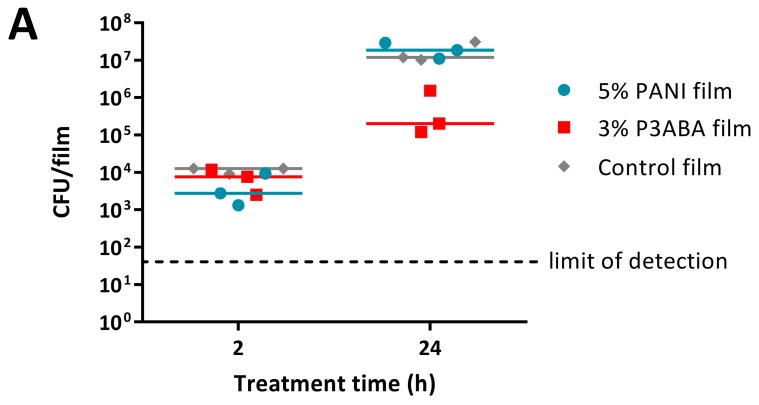

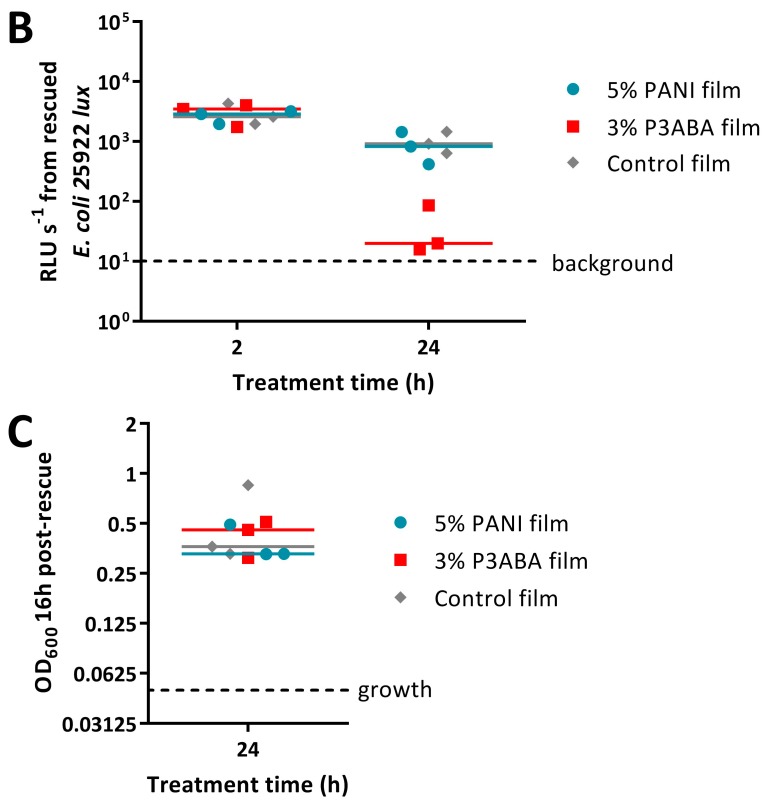
Sensitivity of *E. coli* 25922 *lux* and *S. aureus* 6538 to PANI and P3ABA films. ~10^4^ CFU of *E. coli* (**A**,**B**) or *S. aureus* (**C**) in 10 μL LB broth was sandwiched between two pieces of PANI film, P3ABA film, or control film for 2 h (**A**,**B**) and 24 h (**A**–**C**). The cells were rescued by washing the film samples with LB broth and transferred to a 96 well plate (**B**,**C**). Each point represents the median of three technical replicates and each bar represents the median of each biological replicate. The rescued *E. coli* cells were also enumerated with plate counts (**A**). The cells in the 96 well plate were incubated at 37 °C for 16 h and light release (**B**) or OD_600_ (**C**) was measured. The vertical axes show the viable cell counts (**A**) and bioluminescence measurements (**B**) from the recovered *E. coli* cells, and OD_600_ measurements (**C**) from the recovered *S. aureus* cells, with each data point representing an independent experiment and the line representing the median. The limit of detection for the plate counts is 50 CFU/mL. Background luminescence readings are 10 RLU s^−1^. OD_600_ readings above 0.05 are considered as growth.

PANI and P3ABA films gave no reduction in bacterial viability for *E. coli* 25922 *lux* for after a 2 h challenge, which was indicated by both plate counts and bioluminescence readings (Figure 6A,B). Films containing P3ABA were more active than their PANI counterparts after 24 h exposure, with the former reducing the plate counts and bioluminescence levels by ~2 log relative to the untreated cells (Figure 6A,B). The activity of 3% P3ABA films against *E. coli* after 24 h treatment was statistically significant (2-way RM ANOVA; CFU/mL *p* value: less than 0.05; RLU s^−1^ *p* value: less than 0.05). The similarity in trends seen between the plate counts and bioluminescence measurements from *E. coli* 25922 *lux* that was treated with PANI and P3ABA films confirmed that the bioluminescence-based experimental approach to determining the activity of a non-absorbent surface was appropriate to use for further testing. The results presented show that non-absorbent surfaces containing P3ABA can reduce bacterial load after a 24 h exposure.

Following from this, the optimised protocol for determining the activity of 5% PANI and 3% P3ABA films was used against *S. aureus* 6538 with an increase in OD_600_ above 0.05, indicating the presence of viable cells. *S. aureus* 6538 was treated for only 24 h as PANI and P3ABA films were not active against *E. coli* 25922 *lux* following 2 h treatments (Figure 6A,B) and *S. aureus* was less sensitive than *E. coli* to PANI and P3ABA in suspension (Figure 2). Both 5% PANI in films and 3% P3ABA in films displayed no activity against *S. aureus* 6538 inoculated in LB broth (Figure 6C).

### 2.4. Characterisation of the Action of PANI and P3ABA Films against E. coli and S. aureus

The activity of 5% PANI and 3% P3ABA films against *E. coli* and *S. aureus*, was poorer than expected. We hypothesised that this might be due to inoculating large numbers of bacteria in rich media. To test this hypothesis, films containing PANI and P3ABA were challenged with a range of concentrations of *E. coli* 25922 *lux* and *S. aureus* 6538. The test organisms were washed in saline to simulate a low nutrient environment. A 2 h exposure was used, as this contact time is more effective in disrupting transmission pathways. The influence of the presence of organic matter was examined by challenging PANI and P3ABA films with *E. coli* 25922 *lux* washed in LB broth or in 0.85% saline for 2 h.

**Figure 7:**
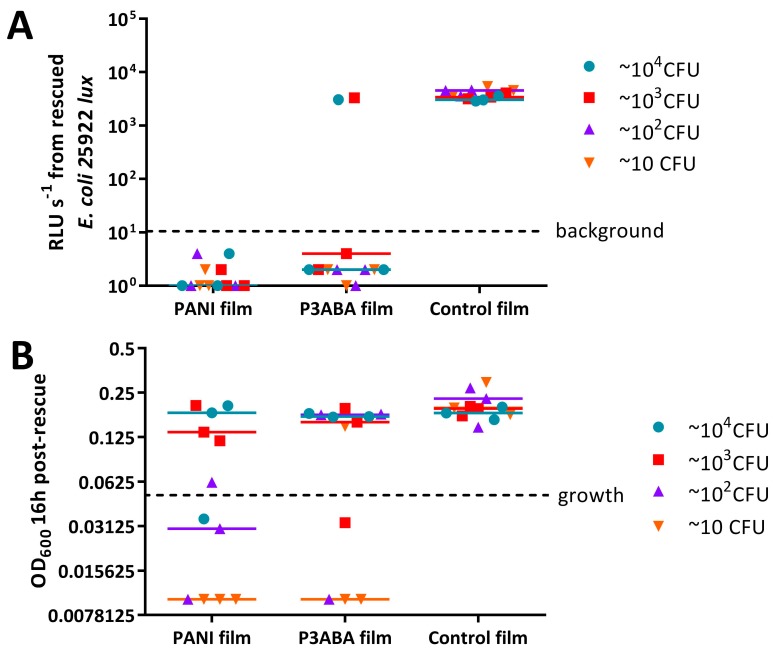
Activity of PANI and P3ABA films against a range of CFU doses of *E. coli* 25922 *lux* and *S. aureus* 6538. ~10 CFU–~10^4^ CFU of *E. coli* (**A**) or *S. aureus* (**B**) in 10 μL 0.85% saline was sandwiched between two pieces of PANI film, P3ABA film, or control film for 2 h. The cells were rescued by washing the film samples with LB broth and transferred to a 96 well plate. The rescued cells were incubated at 37 °C for 16 h and light release (**A**) or OD_600_ (**B**) was measured. The vertical axes show the bioluminescence measurements (**A**) or OD_600_ (**B**) from the recovered cells with each data point representing an independent experiment and the line representing the median. Background luminescence readings are 10 RLU s^−1^. OD_600_ readings above 0.05 are considered as growth.

Bioluminescence from all of the doses of *E. coli* exposed to films containing 5% PANI was reduced to below background levels (Figure 7A). Films containing 3% P3ABA exhibited similar levels of antimicrobial activity (Figure 7A). The antimicrobial activity of PANI and 3PABA films against each inoculum level tested was significantly different from the control film (2-way RM ANOVA, interaction of film type and CFU dose *p* value: less than 0.05). These results indicate that PANI and P3ABA films are active against *E. coli* in low nutrient conditions.

OD_600_ values from ~10^4^ CFU and ~10^3^ CFU doses of *S. aureus* 6538 treated with 5% PANI and 3% P3ABA films were above OD_600_ of 0.05, the threshold for growth, indicating that the films were not active against these higher CFU doses (Figure 7B). The OD_600_ from lower doses of *S. aureus* that was exposed to PANI and P3ABA films did not increase above the threshold for growth implying killing of the inoculated cells occurred (Figure 7B). The activity of PANI films against ~10^2^ and ~10 CFU, and P3ABA films against ~10 CFU was statistically significant (2-way RM ANOVA, film type *p* value: less than 0.05, CFU dose *p* value: less than 0.05). It can be concluded that PANI and P3ABA films are active against low inocula of *S. aureus* in saline.

**Figure 8:**
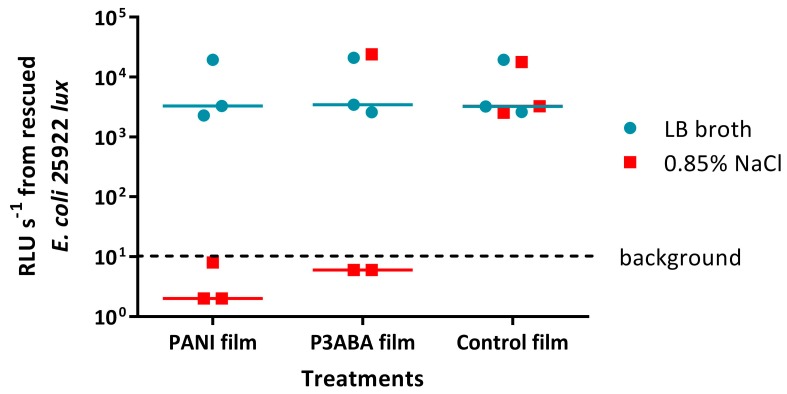
Activity of PANI and P3ABA films against *E. coli* 25922 *lux* in the presence and absence of organic matter. ~10^4^ CFU *of E. coli* in 10 μL LB broth or 10 μL 0.85% saline was sandwiched between two pieces of PANI film, P3ABA film, or control film for 2 h. The cells were rescued by washing the film samples with LB broth and transferred to a 96 well plate. The rescued cells were incubated at 37 °C for 16 h and light release was measured. The vertical axis shows the bioluminescence measurements (RLU s^−1^) from the recovered cells with each data point representing an independent experiment and the line representing the median. Background luminescence readings are ~10 RLU s^−1^.

The effect of the presence of organic matter on the surface activity of films containing PANI or P3ABA was determined by challenging *E. coli* 25922 *lux* in LB broth and 0.85% saline. Bioluminescence levels from *E. coli* 25922 *lux* recovered from 5% PANI films and 3% P3ABA films when inoculated in 0.85% saline were below background levels, whereas *E. coli* 25922 *lux* inoculated in LB broth released the same amount of light as bacteria that were recovered from control films (Figure 8). This indicates that *E. coli* in saline was much more sensitive to PANI and P3ABA films than *E. coli* in LB broth. It is possible that the constituents of LB broth interfere with the contact killing of *E. coli* on films containing PANI and P3ABA.

## 3. Discussion

PANI and P3ABA are promising additives to materials to create contamination resistance surfaces. Factors that may influence the antibacterial efficacy of the surface were explored over short treatment times (up to 4 h). Disrupting transmission pathways through surface decontamination can be best achieved with an antimicrobial agent that kills over a short period of time [71]. The longer a bacterium persists on a surface, the greater the opportunity to be spread [4]. Therefore, rapid decontamination times will decrease the chance that bacteria may be transferred to a new surface before sterilisation is achieved, and will decrease the likelihood of resistance developing [72].

The activity of PANI and P3ABA was determined against *E. coli* and *S. aureus*, representing important pathogens that are found in settings requiring antimicrobial surfaces, such as hospitals and food processing plants. Overall, *E. coli* had greater susceptibility to both PANI and P3ABA in suspension when compared to *S. aureus* (Figure 2). A similar trend was observed for PANI or P3ABA in agar (Figure 4 and Figure 5) and in films (Figure 6). These results demonstrate that PANI and P3ABA are active against the model Gram-negative and Gram-positive bacteria, *E. coli* and *S. aureus*, respectively, in suspension and in different types of surfaces. The differing levels of activity tha were observed against *E. coli* and *S. aureus* highlight how a broad spectrum antimicrobial agent may be more or less effective against a range of bacteria and demonstrates why testing should be done against all the potential target organisms.

The effect of the presence of complex nutrients on the susceptibility of *E. coli* to PANI and P3ABA in suspension was examined. *E. coli* was more susceptible to the antimicrobial action of PANI in suspension when incubated in minimal media when compared to LB broth (Figure 3A). The more efficacious activity of PANI in a low nutrient environment supports the incorporation of this antimicrobial agent in surfaces for applications that are associated with only minor contamination with organic matter. In contrast to this, P3ABA was more active against *E. coli* in rich media relative to minimal media (Figure 3B). The metabolic state of the cell may influence how it responds to bactericidal treatment [73]. The bactericidal action of antimicrobial agents is associated with increased respiration, while bacteriostatic action is characterised by suppressed cellular respiration [73]. The bacteriostatic effect reduces ATP demand and is often the dominant effect blocking bactericidal action [73]. Following from this, if cellular energy output is readily inhibited, such as in cells growing in energy poor conditions, antimicrobial action may result in the inhibition of growth rather than bactericidal killing [73]. Bacterial cells that are highly active may therefore be more susceptible to antimicrobial exposure because of accelerated respiration. The reduced sensitivity of *E. coli* cells to P3ABA in low nutrient conditions could be reflective of a predisposition to the bacteriostatic effect. The greater activity of P3ABA in the presence of nutrients that facilitate bacterial cell growth supports the use of P3ABA in surfaces in settings that are associated with contamination of organic matter, such as surfaces in the vicinity of patients with gastrointestinal infections, which are commonly contaminated with faecal matter containing the bacteria.

The feasibility of using PANI and P3ABA as additives to create antimicrobial surfaces was examined by determining the activity in suspension. Following confirmation of activity against *E. coli* and *S. aureus* (Figure 2), the activity of PANI and P3ABA as agents that are added to absorbent and non-absorbent surfaces was investigated. Overall, both PANI and P3ABA are most active in suspension, followed by in agar and then in films. *E. coli* treated with 0.5% PANI in suspension for 4 h was reduced in numbers by 2 log (Figure 3A), while 1% PANI in agar did not reduce the viable cell count, even after 8 h of treatment (Figure 4A). For surface incorporated PANI to achieve comparable activity to PANI in suspension, a higher concentration is required. This is demonstrated by total knockdown of *E. coli* after a 4 h exposure to 2% PANI in agar (Figure 4A); a result that was achieved by a concentration of 0.5% in suspension (Figure 3A). The reduction in activity of surface incorporated PANI and P3ABA is reflective of how immobilisation in a surface can affect bactericidal activity and how different surface matrixes may influence this in different ways [28].

In this study, the antibacterial activity of PANI and an fPANI were determined by the quantification of the viable cells remaining after a period of challenge, using either classical culture-based techniques, or measuring bioluminescence of genetically modified bacteria as a surrogate measure of viability. Future studies may be enhanced by coupling this type of analysis with scanning electron microscopy (SEM) of bacteria on surfaces and fluorescence microscopy after live/dead staining. SEM has previously allowed for visualisation of bacterial killing by fPANIs to the conclusion that the antimicrobial mode of action eventually leads to a loss of cell integrity [74,75]. Fluorescence microscopy of live/dead stained biofilms has allowed for the activity of another fPANI, polysulfanilic acid, to be followed, with the killing of bacteria being established in biofilms and the release of biomass from the surface, imaged [54]. In the study of bacterial attachment to surfaces real time imaging, e.g., using differential interference contrast microscopy [76] may allow for a better understanding of the interaction of bacteria with surfaces and the factors that influence resistance to colonisation.

It is believed that PANI and P3ABA exert antimicrobial action following contact with a bacterial cell [45,77]. Thus, the reduced contact that occurs between a bacterial cell and surface incorporated PANI and P3ABA (relative to in suspension) would mediate the decrease in antimicrobial efficacy. The least amount of contact between the antimicrobial agent and a bacterial cell would occur for non-absorbent surfaces, which mirrors the decreased activity that was observed for PANI and P3ABA in films. 2% PANI and 2% P3ABA in agar (Figure 5) were able to mediate knockdown of *S. aureus* in 8 h and 15 min, respectively, while 5% PANI and 3% P3ABA in films were unable to reduce bacterial cell numbers after a 24 h treatment (Figure 6C). In this example, higher concentration and treatment time did not ameliorate the reduction of activity for polymers that were incorporated into a non-absorbent surface. The results of this work demonstrate why it is important to test antimicrobial agents, first in suspension (associated with quick and reproducible results) before testing as part of a surface, which should reflect the final application [71].

In real world settings, antimicrobial surfaces may be challenged with a range of inocula. It is well known that the size of the inoculum that is used can influence the magnitude of antimicrobial activity in susceptibility testing [56]. In general, higher inocula need a higher concentration of antimicrobial agent and/or a longer treatment time to achieve knockdown [78]. The surface activity of PANI in film and P3ABA in film was affected by *S. aureus* inoculum size with activity demonstrated only for lower inocula (Figure 7B). The decreased surface activity in the presence of high numbers of bacteria may be mediated by the piling of bacterial cells on top of each other, thereby reducing direct contact with the antimicrobial agent for a portion of the population [79]. The results that are presented demonstrate the necessity to perform antimicrobial surface testing with appropriate inocula to simulate the potential challenges that would occur in the real world application. Surfaces in hospitals are considered to be contaminated when aerobic colony counts exceed 2.5 CFU/cm^2^; however, sampling of objects in patient hospital rooms has demonstrated contamination with a range of bacterial loads (up to 10^4^ CFU/m^2^, equivalent to 10^2^ CFU/cm^2^), including 10^3^ CFU/m^2^ (equivalent to 10 CFU/cm^2^) of MRSA on door handles [80,81,82,83]. Therefore, antimicrobial surfaces in hospitals would need to be active against up to 10^4^ CFU/m^2^ of contaminants to prevent bacterial spread.

Organic soiling of antimicrobial surfaces is a known cause of loss of activity and thus was investigated for surfaces containing PANI and P3ABA [25,71,84]. Surface activity of both PANI in film and P3ABA in film was decreased in the presence of organic matter (Figure 8). Organic matter can interfere with contact between the bacterial cell and the antimicrobial agent—particularly for charged proteins and polysaccharides that can disrupt charge based interactions—thus providing protection from antimicrobial action [28,85,86]. Additionally, contaminating organic matter may inactivate antimicrobial agents [86]. Typical organic contaminants on hospital surfaces include blood and faecal matter [25,71]. It is important that antimicrobial surfaces are tested in conditions, including contamination with organic matter, relevant to the application to verify that the surfaces will be sufficiently active in these settings [25].

While it is not ideal that a reduction in surface activity was observed, the loss of activity upon soiling is common and the effect of organic soiling can be reduced by regular cleaning. Therefore, antimicrobial surfaces need to be able to withstand any adverse environmental conditions that are associated with cleaning [27]. PANI and P3ABA have thermal stability up to 300 °C and environmental stability in the conducting form [45,46,47,48]. An fPANI containing surface was demonstrated to retain activity against *E. coli* and *S. aureus* after 10 repeated challenges if hydrogen peroxide, but not bleach, was the cleaning agent [87]. Future work will include examining the influence of current cleaning procedures on the activity of surface incorporated PANI and P3ABA.

P3ABA containing surfaces demonstrated potential as contamination resistant surfaces for applications. P3ABA as part of a non-absorbent surface reduced *E. coli* by 2 log after a 24 h incubation (Figure 6B), while an absorbent surface containing 2% P3ABA cleared the bacterial load after 15 min (Figure 4B). The P3ABA containing surfaces in this work indicate a superior performance than has been reported for triclosan, a popular additive claiming antimicrobial activity, which had no effect on the viable cell count of *E. coli* following a 24 h exposure [88]. Similarly, triclosan-incorporated plastic only inhibited *E. coli* O157:H7 after a 24 h incubation [89] and triclosan melt-mixed with 4.5% polystyrene inhibited *E. coli* Y 1090 for 5 h, after which the viable cell number increased [90]. Materials containing P3ABA may therefore have a future as a cost-effective antimicrobial surface to prevent or at least reduce the undesirable spread of micro-organisms.

## 4. Materials and Methods

### 4.1. Bacterial Strains and Growth Conditions

*E. coli* ATCC 25922 (referred to as *E. coli* 25922) and *S. aureus subsp*. *aureus* ATCC 6538 (referred to as *S. aureus* 6538) were used in this work because they are routinely used as control organisms to verify that antibiotic susceptibility results are accurate [56,91]. *E. coli* 25922 was tagged with an integrating plasmid (p16S*lux*) containing the bacterial luciferase (*lux*) operon (designated *E. coli* 25922 *lux*) [92,93]. *E. coli* 25922 *lux* was used for testing of surfaces containing PANI and P3ABA [28]. All strains were grown at 37 °C, with 200 rpm agitation where appropriate. The University of Auckland Institutional Biological Safety Committee approved the construction and use of genetically modified Enterobacteriaceae (GMO04-UA0027).

### 4.2. Media and Chemicals

PANI and P3ABA were synthesised via chemical oxidation of aniline and 3-aminobenzoic acid monomers, respectively [45]. Cell biology reagents were purchased from Sigma-Aldrich (New South Wales, Australia). Bacteria were cultured in LB broth (BD) or in minimal media. Minimal A medium was used to support growth in a minimal environment, providing only essential nutrients. A 5× minimal A solution was made according to the following: 5 g (NH_4_)_2_SO_4_, 22.5 g KH_2_PO_4_, 52.5 g K_2_HPO_4_, 2.5 g sodium citrate·2H_2_O. After autoclaving, this solution was diluted to 1× with sterile water and the following sterile solutions, per litre: 1 mL 1 M MgSO_4_·7H_2_O, 0.1 mL 0.5% thiamine plus the carbon source (10 mL of 40% succinate solution per litre).

### 4.3. Preparation of PANI and P3ABA Suspensions

PANI was finely ground using a mortar and pestle. This insoluble powder requires shaking at 200 rpm to stay in suspension. Reflecting the improved solubility of P3ABA, this polymer was suspended in broth by sonication (QSonica Q700 Sonicator, Newtown, CT, USA) at the following settings: amplitude 30, elapsed time 10 s, repeat 4×. Suspensions of PANI and P3ABA were prepared at 1% (*w*/*v*) for a final concentration of 0.5%.

### 4.4. Activity of PANI and P3ABA Suspensions against E. coli and S. aureus

Turbid overnight cultures of test bacteria were diluted to 10^6^ CFU/mL in LB broth (*E. coli* 25922 *lux* and *S. aureus* 6538) or minimal A salts with 0.4% succinate (*E. coli* 25922 *lux*) [94]. The inocula were retrospectively enumerated on LB agar plates [64]. 500 μL of PANI suspension, P3ABA suspension, and growth media (untreated cells) were inoculated with 500 µL of diluted culture. At 0.5 h, 1 h, 2 h, and 4 h time points, each experimental sample was enumerated on LB agar plates. Following incubation, colonies were counted and CFU/mL was calculated. At least three biological replicates were obtained.

Linear regression analysis was used to compare the sensitivity of test strains to PANI or P3ABA suspensions. Specifically, the sensitivity of *E. coli* and *S. aureus* in LB broth to each suspension was compared and the sensitivity of *E. coli* in LB broth and in minimal media to each suspension was compared. Statistical analysis by linear regression was performed using GraphPad Prism software version 6 (GraphPad Software, Inc., La Jolla, CA, USA). Data was graphed in a scatter plot that was generated with viable cell counts post-treatment (CFU/mL) represented on the *y*-axis and time (h) represented on the *x*-axis. Linear regression was used to fit a straight line (regression line) through the data for the categorical factor (strain type or media type) generating the best-fit value of the slope and intercept. An analysis of covariance (ANCOVA) was used to compare the regression lines from the categorical factors to determine if there was a statistically significant difference in sensitivity.

### 4.5. Activity of Absorbent Surfaces Containing PANI and P3ABA against E. coli and S. aureus

Absorbent surfaces containing PANI or P3ABA can be modelled using agar, as drops of liquid containing bacteria will absorb into the agar surface [64]. Molten agar was mixed with varying amounts of PANI or P3ABA, which when left to set created absorbent surfaces containing the antimicrobial agents. PANI or P3ABA were established in agar at 1% and 2%; PANI was also established in agar at 8%. PANI and P3ABA containing absorbent surfaces were set up in triplicate in a 96 well plate by aliquoting 200 µL of each test agar and 200 µL of LB agar (for the untreated control) into individual wells. A turbid culture of test bacteria was diluted to 10^6^ CFU/mL in broth and retrospectively enumerated. All of the test surfaces were inoculated with 10 µL diluted culture, resulting in 10^4^ CFU in each well [55,56]. Agar samples for background readings received 10 µL LB broth.

At specified time points, bacterial cells were rescued in 200 µL fresh media in a 96 well plate [95]. Each type of absorbent surface was tested for the necessary time to achieve knockdown, therefore, highly active surfaces were tested only for the shorter treatment times. *E. coli* 25922 *lux* and *S. aureus* 6538 were challenged with PANI in agar for the following treatment times: 15 min, 30 min, 1 h, 2 h, 4 h, and 8 h. *E. coli* 25922 *lux* was exposed to P3ABA in agar for the following treatment times: 15 min, 30 min, 1 h, and 2 h. *S. aureus* 6538 was exposed to P3ABA in agar for the following treatment times: 15 min, 30 min, 1 h, 2 h, and 4 h. The viability of rescued *E. coli* 25922 *lux* was assessed after 16 h incubation by measuring bioluminescence using the VICTOR X Multilabel Plate Reader (Perkin Elmer, Foster City, CA, USA). The viability of rescued *S. aureus* 6538 was determined by measuring OD_600_ using the µQuant™ Microplate Spectrophotometer (BioTek Instruments, Winooski, VT, USA). Three biological replicates were obtained for each experiment.

The Friedman test was used to analyse the differences between untreated cells and those that were treated with PANI or P3ABA in agar. When a significant difference was identified (*p* value less than 0.05), specific groups were compared to each other using Dunn’s multiple comparison test. Dunn’s multiple comparison test was used to compare the treated and untreated cells at each time point, with a *p* value of less than 0.05 indicating a significant difference. Thus, comparisons were made between each treatment time for every concentration tested to identify a concentration-contact time combination that is associated with significant surface activity.

### 4.6. Activity of Non-Absorbent Surfaces Containing PANI and P3ABA against E. coli and S. aureus

Non-absorbent surface samples were prepared using SEBS films containing 5% PANI or 3% PANI or no additive (control film). The films were hole punched to generate ~5 mm diameter circles that fit into the wells of a 96 well plate. The film samples were disinfected by immersion in 70% ethanol for 10 min and dried in the Herasafe™ KS (NSF) Class II, Type A2 Biological Safety Cabinet (Thermo Scientific, Auckland, New Zealand) [25].

A turbid overnight culture of test bacteria was diluted to 10^6^ CFU/mL in broth and enumerated. The activity of the PANI and P3ABA containing film samples was determined using the MSTA adapted from Japanese Industry Standard (JIS Z-2801) method [28,70]. A piece of film was placed in an empty well, inoculated with 10 µL of diluted culture, and a second piece of the same type of film was placed on top of the inoculum [28,70]. Film samples for background readings received 10 µL of LB broth. The film treatments were established in triplicate. At the specified time point(s) bacterial cells were rescued in 190 µL LB broth in a fresh 96 well plate [95]. The rescued cells were incubated at 37 °C in a sealed container with moist tissue for 16 h and the viability of rescued cells was determined [95]. Three biological replicates were obtained for each experiment.

For *E. coli* 25922 *lux*, cells were rescued after 2 h and 24 h treatments. The viability of cells post-treatment was assessed by using plate counts and measuring bioluminescence. To this end, a 100 µL aliquot of rescued cells was used to enumerate by drop counts and the remaining 100 µL of rescued cells was added to a dark OptiPlate-96 well microtitre plate containing 100 µL of LB broth for the measurement of bioluminescence using the VICTOR X Multilabel Plate Reader. For *S. aureus* 6538, cells were exposed to film treatments for only 24 h and the viability of cells post-treatment was assessed by incubating 200 µL of rescued cells in a 96 well plate for 16 h and measuring OD_600_ using the µQuant™ Microplate Spectrophotometer.

The activity of PANI and P3ABA in films against *E. coli* 25922 *lux* was analysed using a two-way repeated measures analysis of variation (2-way RM ANOVA). For both the plate counts and the bioluminescence data, the 2-way RM ANOVA determined how *E. coli* 25922 *lux* cell number was affected by two factors, treatment time (2 h and 24 h) and film type (PANI in film, P3ABA in film, no additive). A *p* value of less than 0.05 indicates that the cell number was significantly affected by at least one of the factors. When a significant difference was identified, treated cells were compared to the untreated control for each time point using Dunnett’s multiple comparison test with a *p* value of less than 0.05, indicating a significant difference.

The Friedman test was used to analyse the differences between untreated *S. aureus* 6538 cells and those that were treated with PANI or P3ABA in film. The Friedman test is a nonparametric test that compares three or more matched groups—cells treated with 5% PANI in film, 3% P3ABA in film, and control film. A *p* value of less than 0.05 indicates that at least one of the groups differs from the rest. When a significant difference was identified, specific groups were compared to each other using Dunn’s multiple comparison test. Dunn’s multiple comparison test was used to compare the treated and untreated cells at each time point, with a *p* value of less than 0.05 indicating a significant difference.

### 4.7. Characterisation of the Action of PANI and P3ABA Films against E. coli and S. aureus

#### 4.7.1. Challenge of PANI and P3ABA Films with a Range of CFU Doses of *E. coli* 25922 *lux* and *S. aureus* 6538 in Saline

Film punches were prepared and decontaminated, as described above. The MSTA was performed with bacterial challenges (10^4^ CFU, 10^3^ CFU, 10^2^ CFU, and 10 CFU) prepared in 10 µL saline. The 10^6^ CFU/mL culture was enumerated. Following a 2 h treatment, cells were rescued in 190 µL LB broth and incubated in a fresh 96 well plate for 16 h. Viability of bacteria was assessed by measuring bioluminescence for *E. coli* 25922 *lux* and by measuring OD_600_ for *S. aureus* 6538. The activity of PANI and P3ABA in films against a range of CFU doses of *E. coli* 25922 *lux* and *S. aureus* 6538 was analysed using a 2-way RM ANOVA.

#### 4.7.2. Assay to Evaluate the Influence of the Presence of Organic Matter on the Activity of PANI and P3ABA Films against *E. coli* 25922 *lux*

Film punches were prepared and decontaminated, as described above. MSTA was performed with bacterial challenges (10^4^ CFU) in 10 µL saline or 10 µL LB broth. The inocula were enumerated. Following a 2 h treatment, cells were rescued in 190 µL LB broth and incubated in a fresh 96 well plate for 16 h. The viability of rescued cells was determined by measuring the bioluminescence.

### 4.8. Appendix A Methods

#### 4.8.1. Validation of Utilisation of *E. coli* 25922 *lux*

To examine if *E. coli* 25922 *lux* can be used for the testing of surfaces containing PANI and P3ABA, in place of the non-tagged version, the MIC and MBC of both strains were determined [55]. A range of concentrations of PANI and P3ABA in suspension were tested (0.03125–4%). The suspensions were established at 2× the final desired concentration in 500 µL. The insolubility of PANI required each suspension to be set up separately by weighing the powder into 5 mL tubes and adding 500 µL LB broth. P3ABA suspensions were established from a stock solution using a doubling dilution series. 500 µL LB broth was aliquoted to set up an untreated control.

The PANI and P3ABA suspensions were inoculated with 500 µL of 10^6^ CFU/mL of *E. coli* 25922 and *E. coli* 25922 *lux*. The MIC was defined as the lowest concentration of PANI or P3ABA that was able to inhibit the visible growth of test bacteria following a 24 h treatment [55,56]. Tubes that were observed by eye to have no visible growth were selected for MBC testing. For this, 20 µL of the experimental sample was spread onto six LB agar plates [55,56]. The spread plates were incubated at 37 °C for 16 h and the growth on these plates was determined. When countable colonies were present, the CFU/mL of the sample was calculated. The MBC was defined as the lowest concentration of PANI or P3ABA that either totally prevents growth or results in a ≥99.9% decrease in the initial inoculum following subculture on LB agar plates [55,96]. At least three biological replicates were obtained.

#### 4.8.2. Determination of the Limit of Detection for *E. coli* 25922 *lux* and *S. aureus* 6538 Growing in a 96 Well Plate

The limit of detection of *E. coli* 25922 *lux* and *S. aureus* 6538 growing in a 96 well plate was examined by determining the lowest number of cells added to LB broth in a 96 well plate that can grow to detectable levels [95]. This was achieved by serially diluting an overnight culture in triplicate in a 96 well plate by transferring 20 µL of culture into wells containing 180 µL of LB broth. A range of inocula were established from ~10^9^ CFU/mL to ~1 CFU/mL. The overnight culture was enumerated to confirm the cell numbers that were tested. The 96 well plate was incubated at 37 °C for 16 h in a sealed container with a moist tissue [95]. Growth of bacteria was assessed by measuring bioluminescence using the VICTOR X Multilabel Plate Reader for *E. coli* 25922 *lux* and by measuring OD_600_ using the µQuant™ Microplate Spectrophotometer for *S. aureus* 6538.

## 5. Conclusions

PANI and P3ABA both demonstrated bactericidal activity against *E. coli* and *S. aureus* in suspension and as part of an absorbent surface, with greater activity being observed with P3ABA. PANI in films was not active against *E. coli* or *S. aureus,* while P3ABA in films reduced the viability of *E. coli* after a 24 h treatment. The results that are presented in this work support the use of P3ABA to create contamination resistant surfaces.

## Appendix A

### Appendix A.1. Validation of Utilisation of E. coli 25922 lux as a Proxy of E. coli 25922 for Investigation of PANI and P3ABA as Surface Antimicrobial Agents

#### Appendix A.1.1. PANI Has Similar Activity against *E. coli* 25922 and *E. coli* 25922 *lux* While P3ABA Is Less Active against the Latter

For *E. coli* 25922, a *lux*-tagged version was used as the released bioluminescence can be detected and serves as a marker of cell viability [28,92,93]. Utilisation of a bioluminescently-tagged strain is a practical alternative to enumeration by plate counts for future testing of these potential surface additives against slow growing bacteria, such as *Mycobacterium tuberculosis* [63]. *E. coli* 25922 *lux* has a chromosomal insertion of the bacterial luciferase (*lux*) operon (*luxCDABE*) into the 16S locus [93]. A cell expressing the *lux* operon (*luxCDABE*) will be in an altered state compared to the non-tagged version as cellular energy is diverted in order to generate the luminescence [28]. The bioluminescence reaction consumes reduced flavin mononucleotide (FMNH_2_) and a long chain fatty aldehyde, and tetradecanoic acid is diverted from the fatty acid biosynthesis pathway to regenerate the aldehyde substrate [97]. Therefore, it is possible that the *lux*-tagged version of *E. coli* 25922 may have different sensitivities to PANI and P3ABA compared to the non-bioluminescent version.

Following from this, the activity of PANI and P3ABA in LB broth was determined against *E. coli* 25922 and *lux*-tagged *E. coli* 25922. The measure of activity used was the standard minimum inhibitory concentration (MIC) and minimum bactericidal concentration (MBC) [55]. For PANI in suspension, *E. coli* 25922 and the *lux*-tagged version had similar sensitivities (Figure A1). P3ABA had a similar MIC against *E. coli* 25922 and *E. coli* 25922 *lux* (Figure A2); however, the MBC of P3ABA against *E. coli* 25922 (0.125%) was lower than that for *E. coli* 25922 *lux* (1–4%), which was statistically significant (Mann-Whitney test, *p* value: less than 0.05). The difference in activity of P3ABA observed against *E. coli* 25922 and *E. coli* 25922 *lux* may be reflective of the metabolic burden of light production. The *lux*-tagged *E. coli* 25922 may be less susceptible to the bactericidal action of P3ABA over a 24 h treatment time. Overall, these results support the use of *lux*-tagged *E. coli* for testing of PANI and P3ABA.

#### Appendix A.1.2. The Limit of Detection for *E. coli* 25922 *lux* and *S. aureus* 6538 Recovered in a 96 Well Plate Is 100 CFU/mL

Examination of the activity of PANI and P3ABA in surfaces involved recovery of challenged cells in a 96 well plate, which facilitated high-throughput testing of many concentrations and treatment times against one inoculum [95]. Therefore, it was necessary to determine the limit of detection of *E. coli* 25922 *lux* and *S. aureus* 6538 growing in this manner to enable interpretation of surface testing results. The limit of detection was examined by determining the lowest number of cells added to 180 µL of LB broth in a 96 well plate that can grow to detectable levels [95]. The limit of detection is presented as CFU/mL to relate back to the initial inoculum concentration. The absolute number of cells present in the 180 µL volume in the wells would be roughly 5-fold lower than the CFU/mL value.

After a 16 h incubation of *E. coli* 25922 *lux*, bioluminescence 2 log or more above background levels was detected in the wells inoculated with ~10^9^ to ~10^3^ CFU/mL indicating bacterial growth (Figure A3). For the well inoculated with ~10^2^ CFU/mL, the median bioluminescence level was 2 log above background levels; although, two points were only slightly above background levels (Figure A3). Therefore, the limit of detection of *E. coli* 25922 *lux* in a 96 well plate was 100 CFU/mL, which would correspond to ~20 CFU in the well.

After a 16 h incubation of *S. aureus* 6538, growth was detected in wells inoculated with ~10^9^ to ~10^2^ CFU/mL (with growth defined as OD_600_ above 0.05) (Figure A4). The median OD_600_ value from starting inoculum of ~10 CFU/mL was above the threshold for growth; however, two points were below background levels (Figure A4). Therefore, the limit of detection of *S. aureus* 6538 in 96 well plate was 100 CFU/mL, which would correspond to ~20 CFU in the well.
